# RETRACTED: Chung et al. Long-Lasting Exendin-4-Loaded PLGA Nanoparticles Ameliorate Cerebral Ischemia/Reperfusion Damage in Diabetic Rats. *J. Pers. Med.* 2022, *12*, 390

**DOI:** 10.3390/jpm15010011

**Published:** 2024-12-31

**Authors:** Cheng-Hsun Chung, Shiu-Dong Chung, Yu-Hsuan Cheng, Chun-Pai Yang, Chiang-Ting Chien

**Affiliations:** 1 Department of Life Science, School of Life Science, College of Science, National Taiwan Normal University, No. 88, Tingzhou Road, Taipei City 116, Taiwan; 60643024s@ntnu.edu.tw (C.-H.C.); 80943003s@ntnu.edu.tw (Y.-H.C.); 2 Division of Urology, Department of Surgery, Far Eastern Memorial Hospital, New Taipei City 220, Taiwan; chungshiudong@gmail.com; 3 Department of Nursing, College of Healthcare & Management, Asia Eastern University of Science and Technology, New Taipei City 220, Taiwan; 4 General Education Center, Asia Eastern University of Science and Technology, New Taipei City 220, Taiwan; 5 Department of Neurology, Kuang Tien General Hospital, No. 117, Shatian Road, Shalu District, Taichung City 433, Taiwan; 6 Department of Nutrition, Huang-Kuang University, Taichung 433, Taiwan

The Journal retracts the article “Long-Lasting Exendin-4-Loaded PLGA Nanoparticles Ameliorate Cerebral Ischemia/Reperfusion Damage in Diabetic Rats” [1], cited above.

Following publication, concerns were brought to the attention of the Editorial Office regarding inappropriate image modification and duplication between this publication [1] and previously published article [2].

Adhering to our standard procedure, the Editorial Office and Editorial Board conducted an investigation that confirmed overlap of sub-images between Figure 4A published in [1] and sub-images in Figure 7 from [2], the repetition of certain regions within Figure 4C in [1] and their overlap with sub-images from Figure 3 in [2], as well as image duplication within Figure 10 in [1]. While the authors fully cooperated with the Editorial Office during the investigation, they were unable to satisfactorily explain the above mentioned concerns nor provide replacement figures and meet the required quality standards to consider a correction as per the journal’s original image requirements policy (https://www.mdpi.com/journal/jpm/instructions#oriimages). As a result, the Editorial Board has lost confidence in the reliability of the findings and decided to retract this publication [1], as per MDPI’s retraction policy (https://www.mdpi.com/ethics#_bookmark30).

This retraction was approved by the Editor-in-Chief of the *Journal of Personalized Medicine.*

The authors agreed to this retraction.
